# WM–STGCN: A Novel Spatiotemporal Modeling Method for Parkinsonian Gait Recognition

**DOI:** 10.3390/s23104980

**Published:** 2023-05-22

**Authors:** Jieming Zhang, Jongmin Lim, Moon-Hyun Kim, Sungwook Hur, Tai-Myoung Chung

**Affiliations:** 1 Department of Computer Science and Engineering, Sungkyunkwan University, Suwon 16419, Republic of Korea; jieming2021@skku.edu (J.Z.); jm.lim@skku.edu (J.L.); swh500@gmail.com (S.H.); 2 Hippo T&C Inc., Suwon 16419, Republic of Korea; mhkim@skku.edu

**Keywords:** gait recognition, graph convolution network, Parkinson’s disease

## Abstract

Parkinson’s disease (PD) is a neurodegenerative disorder that causes gait abnormalities. Early and accurate recognition of PD gait is crucial for effective treatment. Recently, deep learning techniques have shown promising results in PD gait analysis. However, most existing methods focus on severity estimation and frozen gait detection, while the recognition of Parkinsonian gait and normal gait from the forward video has not been reported. In this paper, we propose a novel spatiotemporal modeling method for PD gait recognition, named WM–STGCN, which utilizes a Weighted adjacency matrix with virtual connection and Multi-scale temporal convolution in a Spatiotemporal Graph Convolution Network. The weighted matrix enables different intensities to be assigned to different spatial features, including virtual connections, while the multi-scale temporal convolution helps to effectively capture the temporal features at different scales. Moreover, we employ various approaches to augment skeleton data. Experimental results show that our proposed method achieved the best accuracy of 87.1% and an F1 score of 92.85%, outperforming Long short-term memory (LSTM), K-nearest neighbors (KNN), Decision tree, AdaBoost, and ST–GCN models. Our proposed WM–STGCN provides an effective spatiotemporal modeling method for PD gait recognition that outperforms existing methods. It has the potential for clinical application in PD diagnosis and treatment.

## 1. Introduction

With the increase in the aging population, age-related cognitive disorders have become more prevalent in recent years. Parkinson’s disease (PD), a common progressive degenerative disease of the central nervous system, is characterized by movement disorders such as muscle stiffness, hand tremor, and slow movement. Early detection of PD is crucial for timely treatment and proper medication.

Gait is an important indicator of health status, and the detection of gait abnormalities can serve as an indication to obtain further medical assessment and treatment. Reference [1] observes that analyzing a patient’s gait could be utilized as a clinical diagnostic tool to help doctors recognize two dementia subtypes, Alzheimer’s disease (AD) and Lewy body disease (LBD). This study distinguished LBD and AD using four key gait features: step time variability, step length variability, step time asymmetry, and swing time asymmetry. Beauchet et al. [2] found that a high mean and coefficient of variation of stride length were characteristic of moderate dementia, while an increased coefficient of variation of stride duration was associated with mild cognitive impairment status. Mirelman A. et al. [3] studied the effect of Parkinson’s disease on gait. They highlighted the gait features unique to Parkinson’s disease. In the early stages of Parkinson’s disease, patients have a slower gait and shorter stride length compared to healthy individuals. These gait changes are common in patients with Parkinson’s disease but are not unique, as many diseases reduce gait speed. However, decreased arm swing and smoothness of movement and increased interlimb asymmetry are more specific to Parkinson’s disease and are usually the first motor symptoms. Gait stiffness and staggering may also occur in later stages.

Clinical gait assessment is a commonly used method for performing gait analysis, which is an assessment performed by a clinician. Specifically, the physician needs to observe the patient’s walking performance and then give a score based on criteria of the Unified Parkinson’s Disease Rating Scale (UPDRS) [4] and Simpson–Angus Scale (SAS) [5]. Moreover, utilizing different types of sensors is a popular method. For example, sensors are embedded in the shoe insoles to measure the pressure of the foot against the ground while walking [6]; inertial measurement units and goniometers are fixed to joints, such as the waist and elbow, to measure the walking speed and acceleration [7]. Moreover, some studies have proposed video-based methods [8,9,10]. For example, reflective markers are attached to diverse locations on the human body. The location and trajectory of the markers are analyzed to provide kinematic information by recording with a digital camera. The Vicon Vantage system [10] requires about 8–14 high-precision cameras to provide accurate 3D motion data for gait analysis.

These existing gait analysis methods either require specialist assessment or particular sensors and equipment. It is too costly to deploy such systems. Furthermore, constructing a specific testing environment and training a team to calibrate the system and manage complex data necessitate substantial investment.

To solve this issue, a convenient, low-cost, and clinically practical method is needed to recognize Parkinsonian gait. In clinical practice, Parkinson’s disease screening, follow-up, regular examination, and evaluation of treatment efficacy can be performed in a way that is easily implemented in a clinical setting and is both feasible and effective for patients. With advancements in computer vision, advanced techniques, such as human pose estimation algorithms, have made remarkable progress. Pose estimation is a process that involves localizing a person’s joints in an image or video, and it has been applied to vision-based gait analysis. Previous work on vision-based gait assessment explored the use of the Microsoft Kinect sensor, thus using the 3D joint position provided by the system to analyze Parkinson’s disease gait [11,12]. However, due to the technical limitations of the Kinect depth sensor, 3D joint positions can only be accurately extracted when the participant is located between (0.5 and 4.5) meters from the sensor, which limits the scenarios that can be widely used [13,14].

Recently, there has been an upsurge of interest amongst researchers in conducting gait analysis on conventional color video, which eliminates the requirement for depth sensors and enables the analysis of whole walking durations using a solitary camera. The emergence of novel computer vision techniques and machine learning algorithms has enabled more robust and automated analysis of video data captured by consumer-grade devices. In particular, advanced human pose estimation libraries, such as OpenPose, Detectron, and AlphaPose, have demonstrated their proficiency in extracting precise 2D joint pixel coordinates from video recordings [15,16,17]. Prior research has investigated the utilization of 2D joint trajectories to compute domain-specific features for the identification of Parkinsonian gait and dyskinesia rating from color videos, as highlighted in Refs. [18,19,20,21]. Moreover, the study conducted by Lu et al. [22] delved into the utilization of 3D joint trajectories extracted from video for predicting gait scores related to Parkinson’s syndrome.

Model training in deep learning requires an extensive amount of data. However, there are various restrictions on medical sample acquisition: video collection is restricted by laws and patient privacy, while clinicians are not sufficiently motivated to record patients walking data. The lack of data hinders the application of deep learning. An alternative approach to obtaining real data is to generate synthetic data [23,24]. For example, random noise can be added to existing data, thus extending the available real data and training deep learning models [25]. Hence, data augmentation may be a valuable tool to overcome the inaccessibility of real data in the medical field [26].

Moreover, the input data in the spatial domain is skeletal data, which can be represented in graphical form, while convolution functions on the time axis can be used to capture temporal features such as joint dynamics (frequency, velocity). Naturally, the spatiotemporal graph convolutional network (ST–GCN) [27] is a well-suited model, as it leverages the inherent graph structure of human skeletons, providing an efficient mechanism for learning directly from joint trajectories. The advantage is that it is no longer necessary to develop and compute engineered gait features from joint trajectories, as ST–GCN can learn to utilize the most significant aspects of gait patterns directly from joint trajectories. ST–GCNs have been effectively combined with human pose estimation libraries to score Parkinsonian leg agility [28]. However, the use of these models to recognize Parkinsonian gait directly on a forward video remains unexplored.

In this paper, we hypothesize that Parkinson’s patients have unique gait features that reflect disease-specific cognitive features and underlying pathology. We focus on developing a novel video-based Parkinsonian gait recognition method, using the skeleton and joint location from pose estimation to extract gait features and detect PD gait. The correct identification of brain damage diseases is very useful for clinicians to design appropriate treatment methods.

The present work offers major contributions in three aspects: (1) We propose to use a novel spatiotemporal modeling method based on skeleton data to recognize Parkinsonian gait; in addition, we construct a graph neural network to capture the topological properties of the human skeleton; (2) We design the weighted matrix with virtual connections to meet the specific demands in gait skeleton modeling and propose a multi-scale temporal convolution network to improve the temporal aggregation capability; and (3) An experiment on the dataset shows that compared to other machine learning methods, the proposed model achieves superior performance.

## 2. Related Work

This section provides a review of related works from two perspectives: gait patterns analysis and Parkinson’s gait analysis using machine learning.

### 2.1. Gait Patterns Analysis

In the gait analysis domain, two main data modalities are commonly employed: sensor-based and vision-based approaches. The promising performance of sensors has drawn interest in their application to gait analysis. Lou et al. [29] developed an in-shoe wireless plantar pressure measurement system with a flexible pressure sensor embedded to capture plantar pressure distribution for quantitative gait analysis. Camps et al. [30] proposed to detect the freezing of gait in Parkinson’s disease patients by using a waist-worn inertial measurement unit (IMU). Seifert et al. [31] used radar micro-Doppler signatures to classify different walking styles. Although the sensor-based approach has demonstrated the ability to reflect human kinematics, the need for specific sensors or devices and their requirement to be worn on the human body have limited their convenience in some applications. The vision-based approaches are more convenient and only require cameras for data collection. Prakash et al. [32] utilized an RGB camera to capture joint coordinates from five reflective markers attached to the body during walking, while Seifallahi et al. [33] employed a marker-less system using Kinect cameras to capture RGB–D data to detect Alzheimer’s disease from gait.

Recently, skeleton data have become a popular choice in gait analysis. Some studies have utilized the Microsoft Kinect camera and its camera SDK to generate 3D skeleton data. For example, Nguyen et al. [34] proposed an approach to predict the gait abnormality index by using the joint coordinates of the 3D skeleton as inputs for auto-encoders and then distinguishing abnormal gaits based on reconstruction errors. Elsewhere, Jun et al. [35] proposed a two-recurrent neural network-based autoencoder to extract features from 3D skeleton data for abnormal gait recognition and assessed the performance of discriminative models with these features. In our study, we propose to extract gait features using the skeleton and joint locations obtained from pose estimation.

### 2.2. Parkinson’s Gait Analysis Using Machine Learning

Researchers have experimented with data collected by various sensors for Parkinson’s disease gait analysis. Shalin et al. [36] utilized LSTM to detect freezing of gait (FOG) in PD from plantar pressure data. The experiment required participants with PD to wear pressure-sensitive insole sensors while walking a predefined, provoking path. Labeling was then performed, and 16 features were manually extracted. The best FOG detection model had an average sensitivity of 82.1% and an average specificity of 89.5%. However, these particular sensors and devices are too costly to deploy. In addition, they need to be operated on in a specific place under the guidance of a professional doctor.

Due to the advances in action recognition [27,37,38,39,40,41], a growing number of researchers have applied it to gait recognition [42,43,44], and several studies have used video-based methods to automatically analyze dyskinesia symptoms in PD patients. Mandy Lu et al. [21] proposed a novel temporal convolutional neural network model to assess PD severity from gait videos, which extracts the 3D body skeleton of the participant and estimates the MDS–UPDRS score. Li et al. [20] extracted human joint sequences from videos recorded by PD patients and calculated motion features using a pose estimation method. Then, they applied random forest for multiclass classification and assessed clinical scores based on the UPDRS and Unified Dyskinesia Rating Scale (UDysRS) [45]. Sabo et al. [19] proposed the utilization of a spatiotemporal graph convolutional network (ST–GCN) architecture and training procedure to predict clinical scores of Parkinson’s disease gait from videos of dementia patients. K. Hu et al. [46] proposed a graph convolutional neural network architecture that represents each video as a directed graph to detect PD frozen gait. The experimental results based on the analysis of over 100 videos collected from 45 patients during clinical evaluation have indicated that the proposed method performs well, achieving an AUC of 0.887.

Based on our literature survey, although several studies have evaluated gait videos of Parkinsonian patients, their focus has primarily been on estimating Parkinson’s severity and detecting frozen gait, while recognizing PD gait versus normal gait from the forward video has yet to be reported. Additionally, traditional engineering solutions have proven insufficient to accurately assess motor function based on videos. To address this limitation, we have developed a novel deep-learning based framework to extract skeletal sequence features from forward videos of PD patients, with the ultimate goal of recognizing Parkinson’s gait.

## 3. Materials and Methods

**Figure 1:**
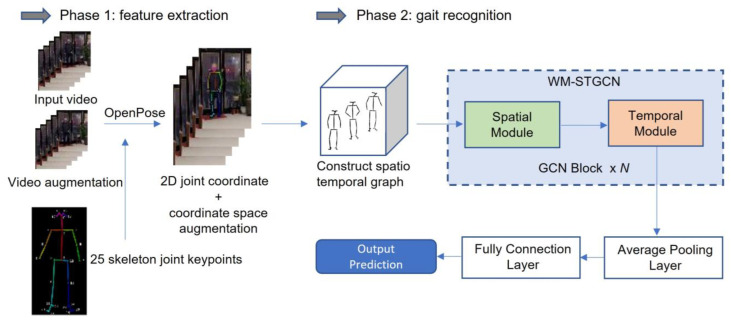
The overall framework of the proposed method.

This part explains our dataset and how the data was preprocessed, and then the model is explained clearly. Figure 1 shows our methodology framework. Our method consists of two phases: feature extraction and gait recognition. Firstly, we augmented the video and then used OpenPose to extract skeleton data. In addition, we augmented the joint coordination space. Secondly, the skeleton data was constructed into a spatiotemporal graph and input to WM–STGCN, and the information in both temporal and spatial dimensions was aggregated by the spatiotemporal graph convolution operation to perform Parkinsonian gait recognition.

### 3.1. Dataset

**Figure 2:**
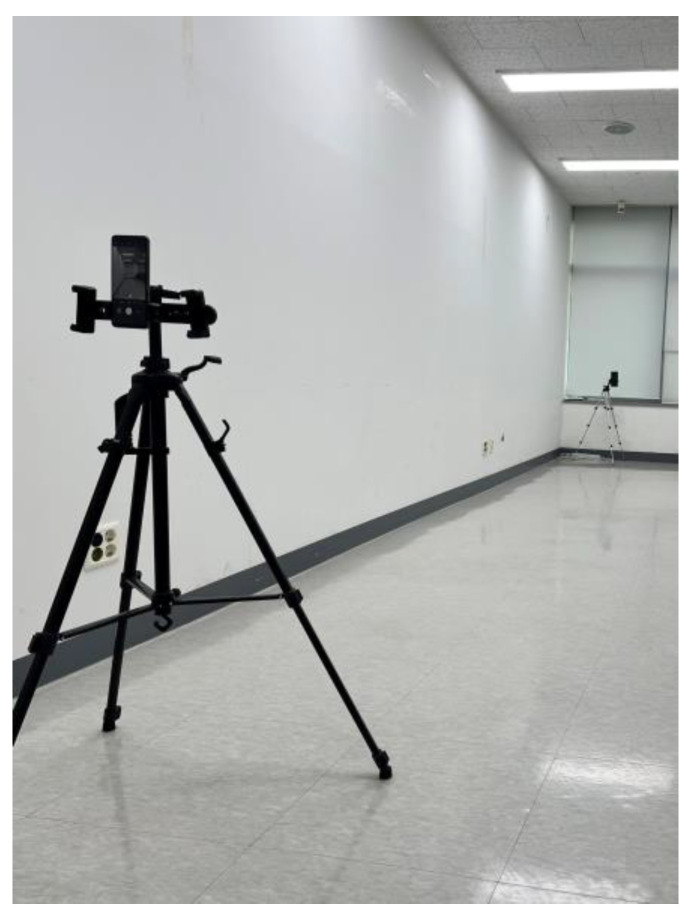
Experiment environment.

**Figure 3:**
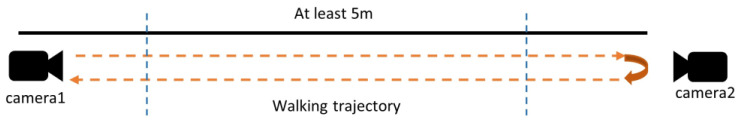
Walking trajectory and camera locations.

We collected the data in an enclosed room for the normal walking video. The wall color was white, with no other colors. The space was 8 m long and 3 m wide, so the cameras could be located. Figure 2 shows the data collection environment. We used two Samsung mobile phones as our recording devices. The video parameters were 1080×1920 pixels at 30 Hz. As depicted in Figure 3, the cameras should be placed in forward of the patient’s walking direction.

Participants wear their comfortable clothes (recommended wear: pants and sweatshirt or T-shirt) and walk straight from beginning to end, then turn around and walk back. During the walk, participants should walk at a normal speed, and for each sequence, the time length is kept to approximately (10 to 20) seconds.

After that, we processed the data to make sure the content was only the frontal view walking. Table 1 lists the collected data details.

We obtained six videos from YouTube for Parkinsonian walking data [47,48,49,50,51,52]. To ensure clarity, their resolution was at least 652×894 pixels, and the frame rate was 30 fps. The video clips of a Parkinson’s patient walking toward the camera without the assistance of others were selected as the data used in our study.

### 3.2. Data Augmentation

**Figure 4:**
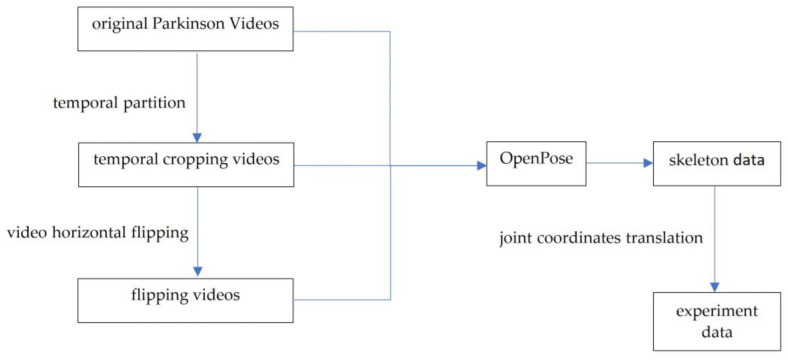
Data augmentation pipeline.

The difficulty in obtaining videos of PD patients walking resulted in a low amount of data. To reduce the class imbalance, we needed to perform data augmentation. Additionally, augmentation can increase the generalization capability of the system. There are two approaches: video augmentation and joint coordinate space augmentation; Figure 4 shows the augmentation pipeline.

We first used temporal partition to crop the original videos, then flipped the video horizontally. After extracting skeleton data, we made joint coordinates space augmentation by translating and adding Gaussian noise.

#### 3.2.1. Video Augmentation

In the video augmentation field, temporal partition and horizontal flipping are two effective tools to augment data on videos.

**Figure 5:**
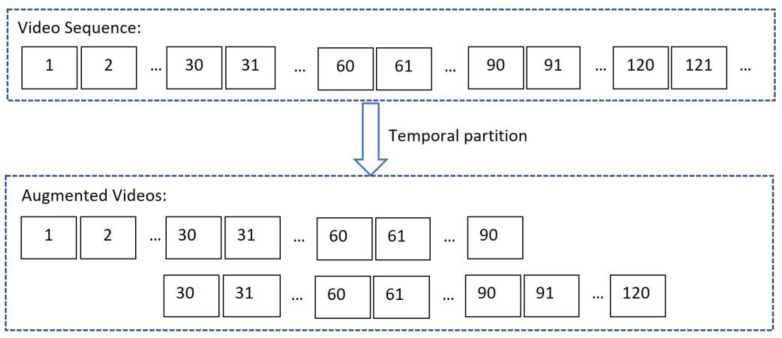
Temporal partition.

We used temporal cropping to implement partition: each video sequence of length l was temporally cropped to a fixed new sequence length k, where k=90 frames, as shown in Figure 5. This allowed a video sequence to be partitioned with an interval of 20 frames. For horizontal flipping, we flipped the entire video to obtain a new video sequence.

#### 3.2.2. Joint Coordinate Space Augmentation

After we extracted skeleton data from videos, a natural idea to augment data is to directly focus on the joint coordinates space. The skeleton data are stored as a dictionary data structure (JSON format files) to allow key and value search to modify the joint value.

**Figure 6:**
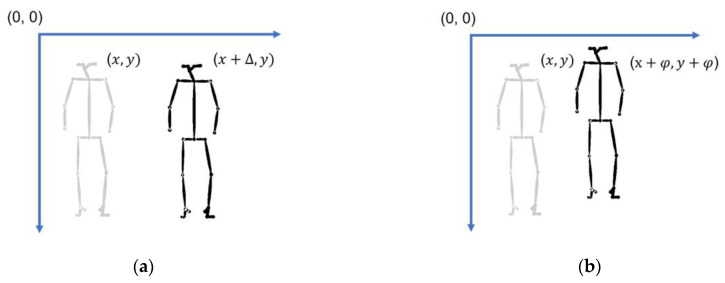
Joint coordinate space augmentation. (**a**) Joint coordinate translation; (**b**) Addition of Gaussian noise to the skeleton data.

We performed the coordinate space augmentation processing in the following two ways:

1. Joint coordinates were translated in the horizontal direction to a new position to allow change in the viewing angle. As shown in Figure 6a, we set the offset Δ=(−0.1, 0.15, 0.2), which means we translated the coordinates of the skeleton data with Δ.
2. Gaussian noise was added to the joint coordinate. Figure 6b shows that the addition of appropriate noise perturbs the skeletal data within a certain range, which allows errors in joint coordinate calculation—for example, interference with the environment, such as background color or cloth texture. We set three Gaussian parameter groups for the experiment for φ(μ,σ), μ=0, σ=(0.01, 0.05, 0.1).

### 3.3. Data Preprocessing

#### 3.3.1. Skeleton Data Extraction

The video sequences are processed to extract 2D skeleton features, where each frame is analyzed using OpenPose, owing to its proficient and robust detection capabilities for 2D joint landmarks in upright individuals. We extract 25 landmarks in the OpenPose-skeleton format, which encompass 2D coordinate values (x, y) and an associated confidence score *c* that indicates the level of estimation reliability.

The key points roughly correspond to body parts: 0: Nose, 1: Neck, 2: RShoulder, 3: RElbow, 4: RWrist, 5: LShoulder, 6: LElbow, 7: LWrist, 8: MidHip, 9: RHip, 10: RKnee, 11: RAnkle, 12: LHip, 13: LKnee, 14: LAnkle, 15: REye, 16: LEye, 17: REar, 18: LEar, 19: LBigToe, 20: LSmallToe, 21: LHeel, 22: RBigToe, 23: RSmallToe, 24: RHeel (L, left; R, right; Mid, middle).

**Figure 7:**
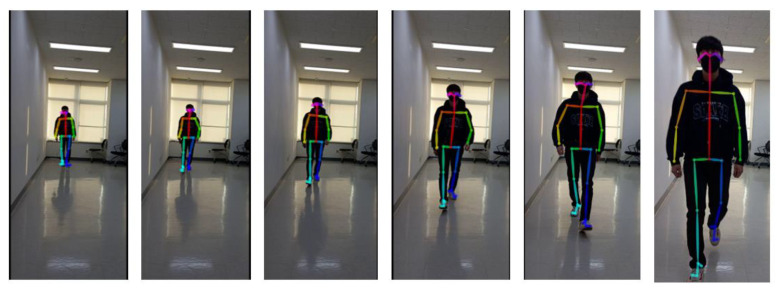
One normal skeleton sequence example.

To obtain sequential key-point coordinate data for each gait sequence, we performed 2D real-time 25-key point body estimation on every image using OpenPose. Figure 7 illustrates the resulting skeleton sequence for a typical normal participant.

#### 3.3.2. Graph Structure Construction

To construct a spatiotemporal graph structure from a sequence comprising N nodes and T frames [27], we employed a pose graph G=(V, E). The node set $V\;=\{v_{t}^{i}\;|\;t=1,\ldots T,\;i=1,\ldots N\}$ denotes the joint positions, where $v_{t}^{i}$ represents the i-th joint at the t-th frame. The feature vector of $v_{t}^{i}$ consists of the two-dimensional coordinate of this joint and the confidence score.

**Figure 8:**
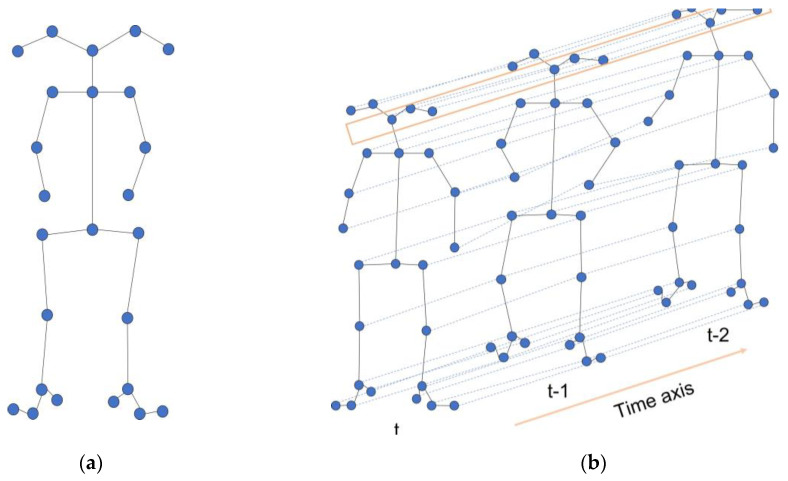
Spatiotemporal graph construction. (**a**) Spatial edges; (**b**) Temporal edges.

The edge set E includes: (a) the intra-skeleton connections, which connect the nodes of each frame according to the connections of human joints, where these edges form spatial edge; Figure 8a shows that we notate it as $E_{s}=\left\{v_{t}^{i}v_{t}^{j}|\left(i,j\right)\in H\right\}$, where H is a set of naturally connected human joints. (b) The inter-frame connections that connect the same joints (nodes) in two consecutive frames, where these edges form temporal edges. Figure 8b shows that we notate it as $\left\{v_{t}^{i}v_{t+1}^{i}\right\}$.

### 3.4. WM–STGCN

#### 3.4.1. WM–STGCN Structure

**Figure 9:**
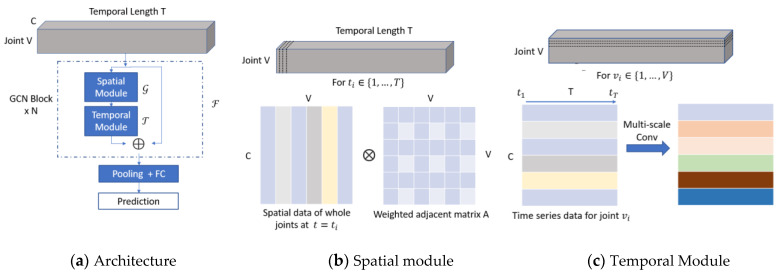
WM–STGCN framework. (**a**) The overall architecture of the proposed network; (**b**) The spatial module leverages the adjacency matrix to fuse features across joints; (**c**) The temporal module employs multi-scale temporal convolutions to capture temporal features.

Figure 9 shows the proposed WM–STGCN model architecture, which takes a sequence of human joint coordinates extracted from gait videos as input and predicts the gait category. Figure 9a provides an overall depiction of the proposed structure, whereas Figure 9b depicts the spatial module, and Figure 9c shows the temporal module.

The whole network comprises *N* GCN blocks (*N* = 9), with output channels of 64, 64, 64, 128, 128, 128, 256, 256, and 256, respectively. A global average pooling layer is added to the back end of the network, and the final output is sent to a SoftMax classifier to obtain the ultimate prediction result. To ensure training stability, residual connections are included in each basic block.

**Figure 10:**
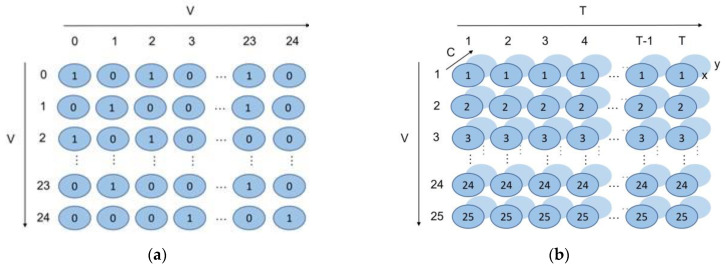
Input data. (**a**) Adjacency matrix A; (**b**) Input feature map of the first GCN block.

Each GCN block ℱ comprises a spatial module G and a temporal module T. The spatial module G combines the features of different joints using sparse matrices derived from the adjacency matrix A, as illustrated in Figure 10a. The output of G is subsequently processed by T to extract temporal features. The computations of ℱ can be summarized as follows:

(1) ℱ(X)=T(G(X,A))+X 

Figure 10b illustrates the input feature map of the first GCN block, wherein a skeleton feature $X\in R^{T\times V\times C}$ is given as input, where T denotes temporal length, V represents the number of skeleton joints, and C signifies the number of channels. Notably, the C input to the first GCN block equals 3.

#### 3.4.2. Spatial Module G: Graph Convolution in the Spatial Domain

In the spatial domain, the convolution of the graph on a certain node $v_{i}$ is defined as follows:

(2) $$f_{out}\left(v_{i}\right)=\sum_{v_{j}\in B_{i}}\frac{1}{Z_{ij}}f_{in}\left(v_{j}\right)\cdot \omega \left(l_{i}\left(v_{j}\right)\right)$$

where $f_{in}$ and $f_{out}$ represent the input and output feature maps, respectively; $v_{i}$ represents a particular node in the spatial dimension; $B_{i}$ represents the sampling area for the convolution of that node (in this work, $B_{i}$ is the 1-neighbor set of $v_{i}$); Z is the normalizing term, which equals the cardinality of the corresponding subset; and w represents the weight function that provides the weight matrix.

We divided the neighborhood B into three subsets of self-connection, physical connection, and virtual connection, and different labels can be assigned to each subset. We discuss the virtual connection in Section 3.4.3. Here, $l_{i}$ is a mapping function: $l_{i}\left(v_{j}\right)\to \left\{0,\ldots ,K,\left(K=3\right)\right\}$, which maps a node in the neighborhood to its subset label.

**Figure 11:**
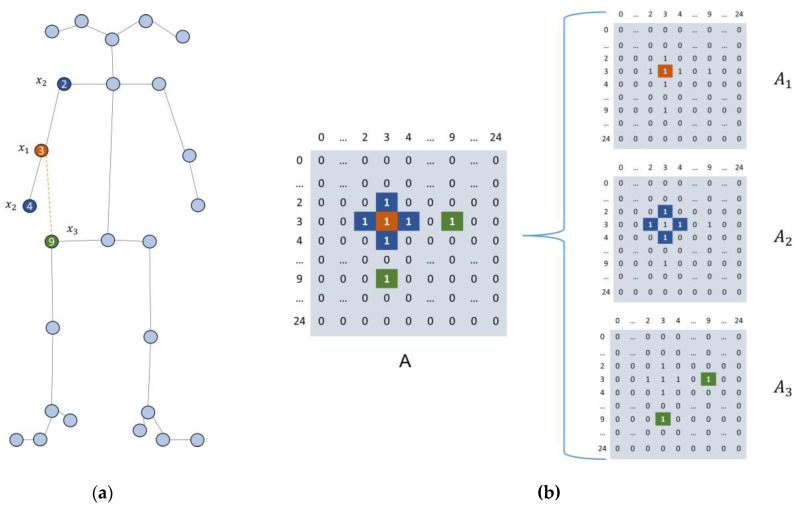
(**a**) A graph of the input skeleton sequence; (**b**) The three submatrices.

Figure 11a shows a graph of the input skeleton sequence, and $x_{1}$ represents the root node itself (orange), $x_{2}$ represents the physically connected node (blue), and $x_{3}$ represents the virtually connected node (green). We use node 1 as the root node of this convolutional computation to explain the mapping strategy. Nodes 2, 4, 9 are its sampled neighboring nodes, which form the neighborhood B, where node 9 provides a virtual connection. Accordingly, as shown in Figure 11b, the adjacency matrix of node 3 is divided into three submatrices $A_{k}$, but ensure that where $A=\underset{k}{\sum }A_{k},\;k=1,2,3$.

Simplifying and transforming Equation (2), the spatial graph convolution can be implemented using the following:

(3) $${Ϝ}_{out}=\sum_{k}\omega_{k}\left({Ϝ}_{in}A_{k}\right),\;k\;=\;3$$

(4) $$A_{k}=\Lambda_{k}^{-\frac{1}{2}}{\bar{A}}_{k}\Lambda_{k}^{-\frac{1}{2}}$$

where, k in Equation (3) represents the amount of convolutional kernel, which is 3 according to the mapping strategy; $A_{k}$ is an N × N normalized adjacency matrix; $\Lambda_{k}^{-\frac{1}{2}}$ is a normalized diagonal matrix. ω is a 1 × 1 convolution operation, which represents the weight function in Equation (2).

In the spatial domain, the input is represented as $G_{in}\in R^{T\times V\times C_{in}}$; upon applying the spatial graph convolution, the resulting output feature map is denoted $G_{out}\in R^{T\times V\times C_{out}}$.

#### 3.4.3. Weighted Adjacency Matrix with Virtual Connection

The spatial structure of the skeleton is represented by an artificial, predefined adjacency matrix, which represents the a priori knowledge of the connections of the human skeleton. However, it cannot generate new connections between non-adjacent joints during training, which means that the learning ability of the graph convolutional network is limited and that such an adjacency matrix is not an optimal choice.

To address the above problems, we design a novel adjacency matrix, which has the following two features:

Virtual connection. We combined some unique features of Parkinson’s gait compared to normal gait (including small amplitude of arm swing, fast frequency and small stride length of foot movement, and random little steps) and introduced some virtual connections, i.e., unnaturally connected joints.

Weighted adjacent matrix. We used a scalar to multiply with the original adjacency matrix to get a new adjacency matrix, which makes distinct kinds of joints with different weights.

With these new designs, we make it possible to generate connections between non-adjacent joints, and give different weights for physical connections, virtual connections and self-connections. We design a new adjacency matrix and obtain a skeletal space structure that is more suitable for describing the Parkinson samples, thus enabling better gait recognition. Specifically, $a_{ij}$ is a scalar:

(5) $$a_{ij}=\{\begin{array}{l} \alpha ,if\;i=j\\ \beta ,if\;joint\;i\;and\;joint\;j\;are\;connected\;physically\\ \gamma ,if\;joint\;i\;and\;joint\;j\;are\;connected\;virtually \end{array}$$

**Figure 12:**
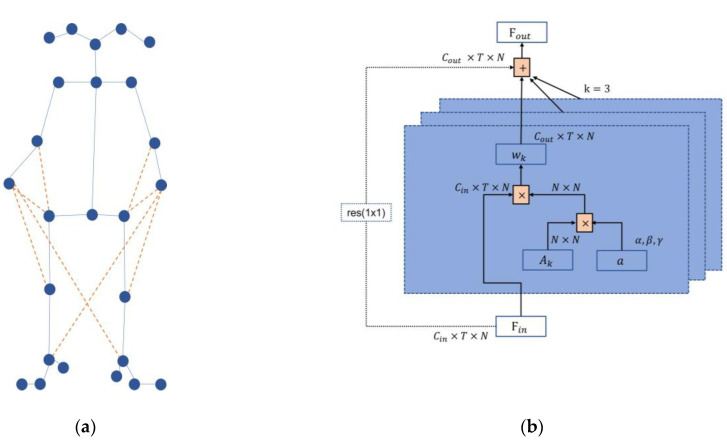
(**a**) Virtual connection; (**b**) Diagram of the graph convolutional layer with weights.

If we set the value of $a_{ii}=0$, this indicates that we eliminate the self-connection of each joint. Additionally, we distinguish between physical and virtual dependencies between joints. The physical dependency, represented by β and depicted as blue solid lines in Figure 12a, captures the intrinsic connection between joints. The virtual dependency, depicted as orange dashed lines in Figure 12a, represents the extrinsic connection between two joints, which is also crucial for gait recognition. We use the parameter γ to model this virtual relationship. For example, although the left hip and left hand are physically disconnected, their relationship is essential in identifying Parkinsonian gait.

After adding weights, the graph convolution formula in spatial dimension can be transformed from Equation (3) to the following:

(6) $${Ϝ}_{out}=\sum_{k}\omega_{k}{Ϝ}_{in}\left(A_{k}\cdot a\right)$$

Figure 12b shows the process of weight addition, where the adjacency matrix of each layer consists of $A_{k}$ and weight $a_{\;}$ together, k denotes the number of subsets, and the dashed line indicates that the residual convolution operation is required only when $C_{in}$ is different from $C_{out}$.

**Figure 13:**
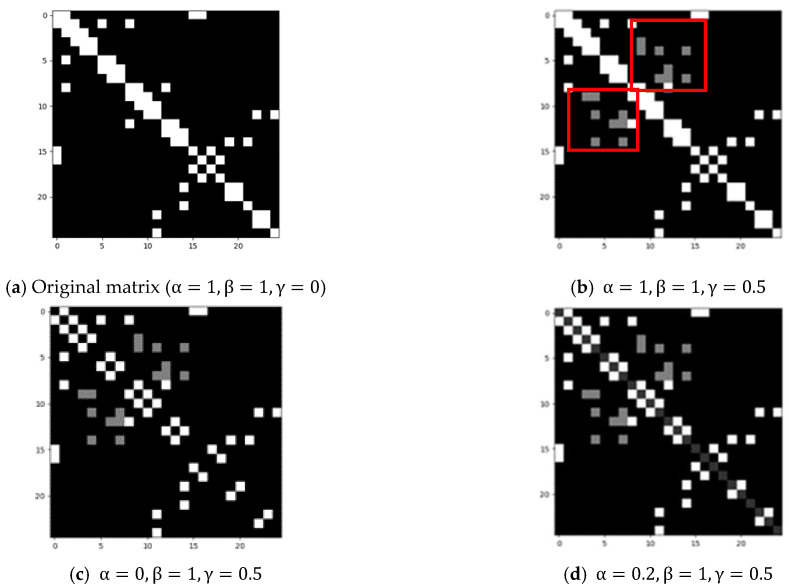
Different parameters for weighted matrix.

For the experiment, we tested 4 cases: ① α=1, β=1, γ=0; ② α=1, β=1, γ=0.5; ③ α=0, β=1, γ=0.5; ④ α=0.2, β=1, γ=0.5. This means that we tested the performance of the model with self-connection, 0.5 weight virtual connection, without self-connection and 0.2 weight self-connection and 0.5 weight virtual connection. Figure 13 shows the corresponding weighted adjacency matrixes. The red box marks the representation of the virtual connection in the matrix.

#### 3.4.4. Temporal Module T: Graph Convolution in Temporal Domain

**Figure 14:**
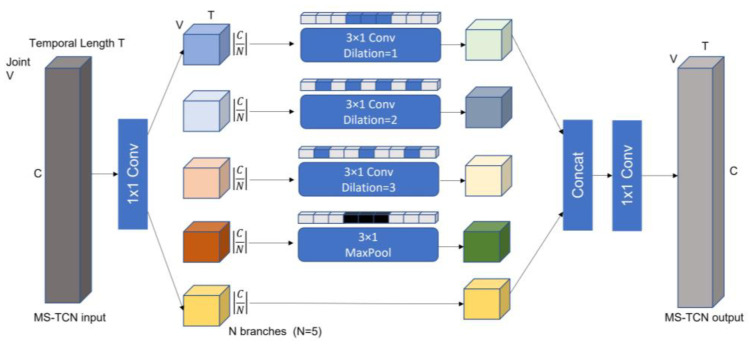
Multi-scale temporal convolution network.

G captures the spatial dependencies between adjacent joints, and to model the temporal changes of these features, we employed a multi-scale temporal convolution network (MS–TCN). Unlike many existing works that employ temporal convolution networks with fixed kernel sizes $k_{t}\times 1$ throughout the architecture, we designed a MS–TCN, as shown in Figure 14, to promote the flexibility and temporal modeling capability by using multi-group convolution.

The adopted multi-scale TCN contains five branches: a 1 × 1 convolution branch, a Max-pooling branch, and three temporal convolutions with kernel size 5 and dilations from (1 to 3). Every branch contains a 1 × 1 convolution, which is used to reduce channel dimension before the expensive convolution 3 × 1. Additionally, the 1 × 1 convolution introduces additional nonlinearity via a nonlinear activation function, thereby increasing the network’s complexity, and enabling it to be deeper. This output continues to be fed into the spatial graph convolution, as shown in Figure 9, and it is fed into the fully connected layer only in the last GCN block.

The MS–TCN enhances vanilla temporal convolution layer’s receptive fields, and improves the temporal aggregation capability. At the same time, it reduces computational cost and parameters through reduced channel width for each branch.

## 4. Experiments

### 4.1. Implementation Details

We used NVIDIA GeForce RTX 2080Ti GPU with 12 GB memory, Intel(R) Core(TM) i9-10900 CPU with 2.80 GHz 64 GB RAM to build the deep learning framework using PyTorch in Windows 10 environment. We used CUDA, Cudnn, OpenCV, and other required libraries to train and test the Parkinsonian gait recognition model. The batch size during training and testing was 16. The base learning rate was 0.1. We chose SGD as optimizer with step [20,30,40,50]. Following data preprocessing, we obtained 160 normal samples, and 150 Parkinsonian samples. We split our dataset into a training set and a test set, with a ratio of 80% and 20%, respectively. The test set comprised 32 normal samples and 30 Parkinsonian samples.

### 4.2. Evaluation Metric

In this study, we defined Parkinsonian gait samples as positive and normal gait samples as negative. We utilized widely accepted evaluation metrics, including True Positive (TP), False Negative (FN), False Positive (FP), and True Negative (TN), to accurately classify samples into these categories. To evaluate the performance of our method, we selected accuracy, precision, sensitivity, specificity, false alarm, miss rate, and F1 score as our evaluation metrics. A higher value for accuracy, precision, sensitivity, specificity, and F1 score indicates better model performance. In contrast, a smaller value for false alarm and miss rate indicates better performance.

Accuracy reflects the ability of the model to correctly judge the overall sample, i.e., the ability to correctly classify Parkinsonian samples as positive, and normal samples as negative.

Precision reflects the ability of the model to correctly predict the positive samples, i.e., how many of the predicted Parkinsonian samples are true Parkinsonian samples.

Sensitivity is defined as the proportion of Parkinsonian samples predicted to be Parkinsonian samples to the total number of Parkinsonian samples. Specificity reflects the proportion of normal samples that are predicted as normal samples to the total normal samples.

(7) $$accuracy=\frac{TP+TN}{TP+TN+FP+FN}\;$$

(8) $$precision=\frac{TP}{TP+FP}\;$$

(9) $$sensitivity=TPR=\frac{TP}{TP+FN}\;\;$$

(10) $$specificity=TNR=\frac{TN}{FP+TN}\;\;$$

False alarm, also known as false positive rate or false detection rate, is obtained by calculating the proportion of normal samples predicted as Parkinsonian samples to the total normal samples. Miss rate is obtained by calculating the proportion of Parkinsonian samples that are predicted as normal samples to the total Parkinsonian samples.

(11) $$false\;alarm=FPR=\frac{FP}{FP+TN}\;$$

(12) $$miss\;rate=FNR=\frac{FN}{TP+FN}\;$$

Furthermore, *F*1 score is widely used in model evaluation. This is the harmonic mean of the precision and recall, which can reflect the performance of the model in a balanced way.

(13) $$F1\;score=2\times \frac{Precision\times Recall}{Precision+Recall}$$

### 4.3. Results and Discussion

**Figure 15:**
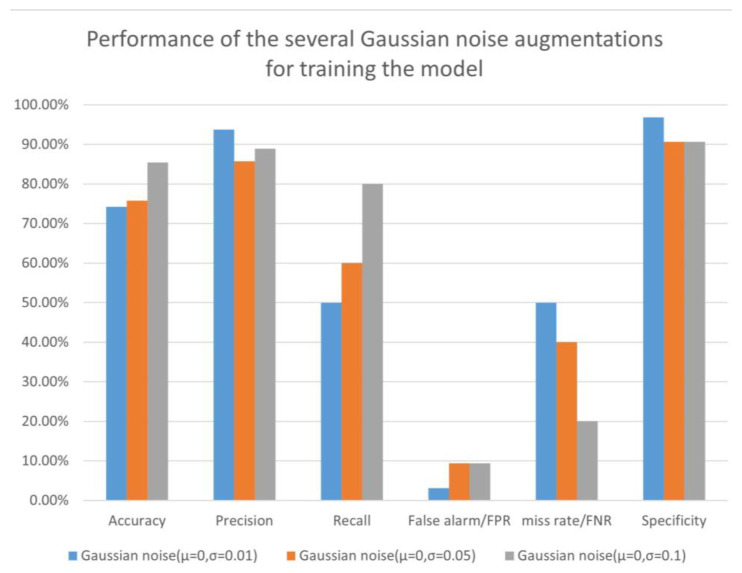
Performance of the several Gaussian noise augmentations.

**Figure 16:**
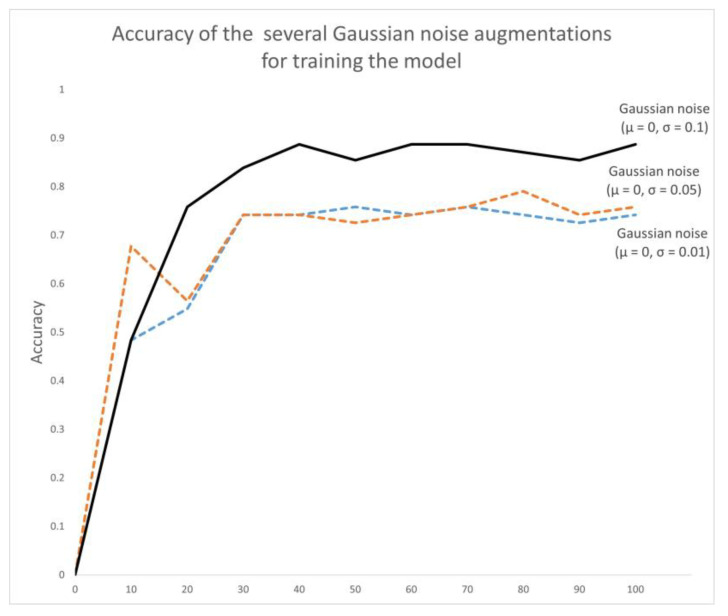
Accuracy of the several Gaussian noise augmentations.

We experimented with different parameters of Gaussian noise augmentation with μ=0, and σ=(0.01, 0.05, 0.1). In Table 2 and Figure 15, the experimental results show that the model had the highest accuracy of 85.48% for σ=0.1. Although the precision was 4.87% lower compared to the group with σ=0.01, the sensitivity increased from 60% to 80%, improving the performance of predicting Parkinsonian samples as positive, which was the best of the three experimental groups. Meanwhile, the miss rate was only 20%, which was much lower than the 40% at σ=0.05. Overall, the model showed the best performance for detecting Parkinson’s samples at σ=0.1. Figure 16 shows the accuracy during training based on several groups of Gaussian noise.

For the different weight adjacencies, we tested four cases. When α=1, β=1, γ=0, which is the original matrix containing only self-connections and physical connections. In Table 3, the experimental results showed that the accuracy reached 72.58%, and the recognition miss rate of Parkinson’s gait was 46.67%, the lowest among the four groups. When adding γ=0.5, i.e., 0.5 weight of virtual connections, we found that although the accuracy rate decreased slightly from 72.58% to 70.97%, the sensitivity and miss rate increased.

After removing the self-connection, we found that the accuracy increased by 14.51% and sensitivity increased by 23.33%, while the miss rate decreased from 43.33% to 20%. This indicates that removing the effect of joint self-connection aids the correct recognition of gait.

**Figure 17:**
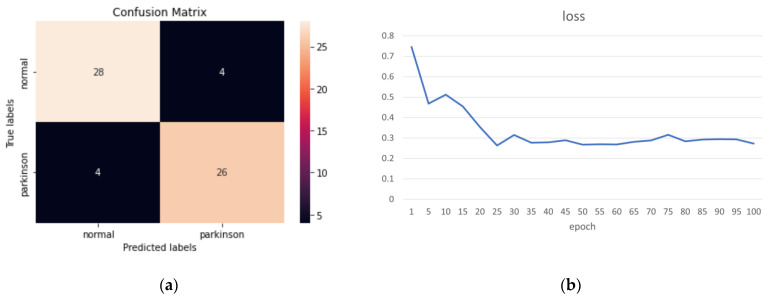
(**a**) Confusion matrix; (**b**) Loss function.

Finally, we achieved the best results with 0.2 weight of the joint self-connections and 0.5 weight of the virtual joint, where the accuracy was 87.10%, the sensitivity was 86.67%, and the miss rate was the smallest, at 13.33%. Figure 17a and Figure 17b show the confusion matrix and loss function, respectively.

Through our experiments, our best result showed an accuracy of 87.10%. Table 4 compares the performance with the other well-known machine learning models of LSTM, KNN, Decision Tree, AdaBoost, and ST–GCN. In particular, Lstm-layer1 means a one-layer network, layer2 means a two-layer network, and the weak learner model in the AdaBoost classifier is 50 decision trees of depth 1.

We conducted an analysis to investigate the superior performance of WM-STGCN in comparison to other models based on the following factors. The first factor is the utilization of a weighted adjacent matrix with virtual connections. The weighted adjacency matrix with virtual connections plays a crucial role in WM–STGCN. While an adjacency matrix without weights can be used to represent adjacency information, a weighted adjacency matrix allows for a more sophisticated representation of adjacency information. Moreover, weights can reflect the structure of the graph in a more granular way, for example, by adjusting weights based on the connection types to emphasize relationships with physical connections or virtual connections. Therefore, using a weighted adjacency matrix enables WM–STGCN models to reflect more detailed graph structures and make better predictions. The second factor is the integration of a multi-scale temporal convolutional network. The multi-scale temporal convolutional network used in this study can enhance the receptive field of temporal convolution, improve time aggregation ability, and extract features from various time intervals. At the same time, it can reduce the computational cost and parameters by reducing the channel width of each branch. Finally, we use a separately designed data augmentation method for both raw video and skeletal data, which also effectively improves the performance of the model.

These advantages enable effective recognition of Parkinson’s disease from gait data. However, there are also some shortcomings. For example, due to equipment limitations, we focused on the RGB color video of the front view, but users cannot guarantee to record high-quality video when using it, which will affect the recognition accuracy. At the same time, our model performance can be further improved by using multi-modal analysis methods, such as adding sensor data. In the future, our WM–STCGN model is expected to be applied to research on gait-related diseases in the elderly, including not only Parkinson’s disease but also dementia, stroke, and other related conditions.

## 5. Conclusions

In this paper, we proposed a novel spatiotemporal modeling approach, known as WM–STGCN, which employs a weighted adjacent matrix with virtual connections and multi-scale temporal convolutional networks to recognize Parkinsonian gait from forward walking videos. Our experimental results demonstrated the effectiveness of the proposed method, which outperformed the machine learning-based methods such as LSTM, KNN, Decision Tree, AdaBoost, and ST–GCN. This method could provide a promising solution for PD gait recognition, which is crucial for the early and accurate diagnosis of PD. We believe that our method can be further improved by integrating it with other advanced deep learning techniques and can be extended to the fields of healthcare and biomedicine.

## Figures and Tables

**Table 1:** Details of the collected data.

Type	Normal
Number of Participants	50
Mean height	174.6 cm
Resolution	1080 × 1920 pxl
Frame rate	30 fps
Length of sample video	10–20 s
Steps of sample video	6–8 steps

**Table 2:** Different parameter results.

Group	Accuracy	Precision	Sensitivity	Specificity	False Alarm/FPR	Miss Rate /FNR
Gaussian noise (μ=0, σ=0.01)	74.19%	93.75%	50.0%	96.87%	3.12%	50.0%
Gaussian noise (μ=0, σ=0.05)	75.81%	85.71%	60.0%	90.62%	9.37%	40.0%
Gaussian noise (μ=0, σ=0.1)	85.48%	88.88%	80.0%	90.62%	9.37%	20.0%

**Table 3:** Results of different weight parameters.

Weight Parameters	Accuracy	Precision	Sensitivity	Specificity	False Alarm/FPR	Miss Rate /FNR
Original (α = 1, β = 1, γ = 0)	72.58%	84.21%	53.33%	90.62%	9.38%	46.67%
α = 1, β = 1, γ = 0.5	70.97%	77.27%	56.67%	84.38%	15.63%	43.33%
α = 0, β = 1, γ = 0.5	85.48%	88.88%	80.0%	90.62%	9.38%	20.0%
α = 0.2, β = 1, γ = 0.5	87.10%	86.67%	86.67%	87.50%	12.50%	13.33%

**Table 4:** Comparison with other models.

Methods	Accuracy	Precision	Sensitivity	F1 Score	Specificity	False Alarm /FPR	Miss Rate /FNR
Lstm-layer1	82.25%	85.19%	76.67%	0.8679	87.5%	12.5%	23.33%
Lstm-layer2	69.35%	100%	63.33%	0.7755	100%	0%	36.67%
KNN	83.87%	85.71%	80%	0.8276	87.5%	12.5%	20%
Decision tree	79.03%	81.48%	73.33%	0.8461	84.38%	15.63%	26.67%
AdaBoost	75.81%	85.71%	60%	0.7059	90.63%	9.38%	40%
ST–GCN	77.42%	90%	56.25%	0.72	93.75%	6.25%	40%
Proposed method	87.10%	86.67%	86.67%	0.9285	87.5%	12.5%	13.33%

## Data Availability

The data presented in this study are available on reasonable request from the corresponding author.
